# Supplementary figures and images for: Machine Learning Based Modelling of Human and Insect Olfaction Screens Millions of compounds to Identify Pleasant Smelling Insect Repellents
Source: bioRxiv. 2026 Mar 14:2023.12.25.573309. Preprint. [Version 2] doi: 10.1101/2023.12.25.573309 (PMC13060945; doi:10.1101/2023.12.25.573309)

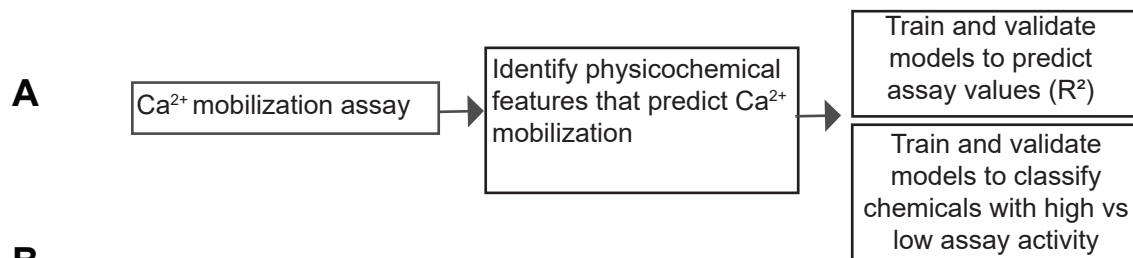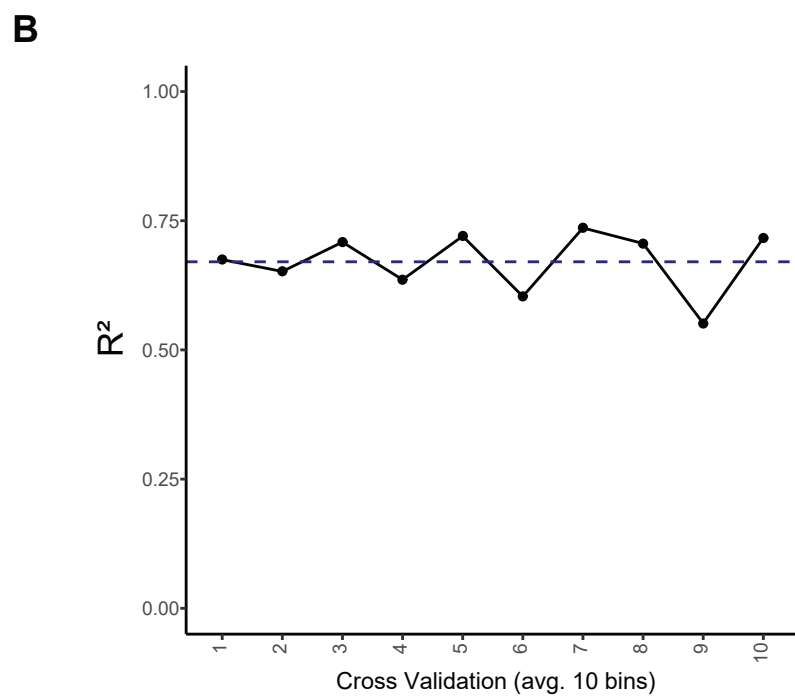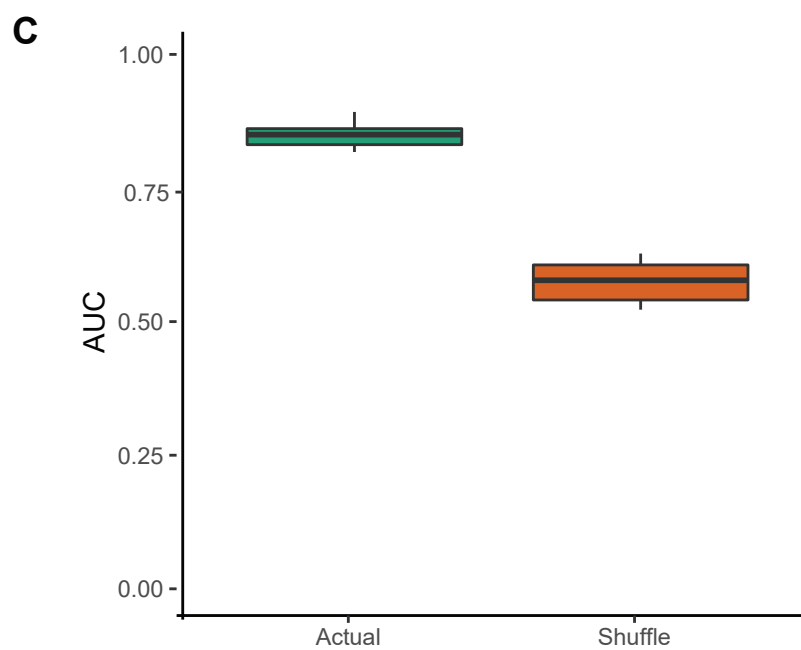

**Figure S1**

Supplement: Supplement 1 — Supplemental Figure S1 A, Overview of the approach to generate and validate the machine learning models. Here, a model-averaged prediction is made, where each model has access to different physicochemical features and is trained on different combinations of training set chemicals. B, The R2 values over the 100 train/test splits, averaged into 10 bins. C, The average classification success is reported over 100 train/test splits, assessed by the area under the ROC curve. Active labels (positive cases) were assigned as chemical scoring in the top ~40% of Ca2+ values. This is compared to shuffling the labels before training, reported as “Shuffle.” [file media-1.pdf]
